# Stakeholder collaboration for solid waste management in a small tourism island

**DOI:** 10.1371/journal.pone.0288839

**Published:** 2023-07-26

**Authors:** Jarotwan Koiwanit, Viachaslau Filimonau

**Affiliations:** 1 Department of Industrial Engineering, School of Engineering, King Mongkut’s Institute of Technology Ladkrabang, Bangkok, Thailand; 2 School of Hospitality and Tourism Management, University of Surrey, Stag Hill, Guildford, The United Kingdom; National Taiwan Normal University - Gongguan Campus, TAIWAN

## Abstract

Although stakeholder collaboration is key for sustainable development of tourism in small islands, research on its determinants is only emerging. The lack of empirical studies hampers an understanding of how effective stakeholder partnerships for sustainability in small tourism islands can be formed and sustained. To partially address this knowledge gap, this study explores stakeholder collaboration for solid waste management in the island of Koh Phayam, Thailand, from the perspective of stakeholder theory, social capital and proximity effect. Semi-structured interviews (n = 26) reveal a lack of understanding of collaboration benefits alongside leadership and reciprocity among stakeholders. However, due to geographical proximity, the level of stakeholder trust in each other is significant, thus indicating potential for future successful partnerships. For these partnerships to become effective, a system of financial incentives for stakeholders to separate and recycle solid waste in situ should be designed. To improve stakeholder communication and reciprocity, capacity building workshops and round tables can be organised. Municipal authorities should lead on solid waste management, and a steering committee comprising the representatives of all other stakeholders needs to be established to oversee the work of municipal authorities. Lastly, the feasibility of setting private public partnerships for solid waste management in Koh Phayam should be considered given the significant extent of knowledge and trust among local stakeholders. External stakeholders, such as farmers, can be involved in management of organic waste, thus extending the scope of partnerships for sustainability beyond the island.

## Introduction

Tourism has significant environmental externalities that should be mitigated towards sustainable development goals [1]. Without mitigation, by 2050, global tourism will consume 154% more energy and 152% more water, and generate 251% more solid waste [2]. Empirical research is required to mitigate the environmental externalities of tourism [3]. This research should measure the scale of environmental impacts associated with tourist activities in different destinations to identify priority areas for mitigative interventions [4]. Research should also understand how tourism stakeholders can be engaged in mitigation [5].

The figures from UNEP [2] highlight solid waste as the key challenge for sustainable tourism development. Tourism enterprises conserve energy and water as this conservation is financially beneficial [6]. For example, by controlling in-room temperature, hotels can save up to 58% in energy cost [7]. In contrast, tourism enterprises do not always associate solid waste management with direct financial benefits [8]. Payment for solid waste is usually based on collection frequency or volume and weight; this payment is however considered affordable [9]. For sustainable development, tourism enterprises should manage solid waste more effectively [10].

The challenge of solid waste management in tourism is pronounced in small islands [11] as remoteness and size hamper their ability to conserve the environment [12]. For example, Wang and colleagues [13] suggest that tourists in small islands generate up to 3.9 kg of solid waste per day. This is almost three times more than the amount of solid waste produced by an average EU resident i.e., 1.4 kg per day [14]. Concurrently, small island destinations have fragile ecosystems with limited carrying capacity [15]. Ineffective management of solid waste damages the natural environment of small islands, thus not only endangering tourism, but also threatening local livelihoods [16].

Research on solid waste management in small island destinations is evolving but significant knowledge gaps persist [13]. One is attributed to the insufficient understanding of the determinants of stakeholder collaboration for environmental conservation [17]. Remoteness and small size of islands suggest limited resources which are prioritised by local stakeholders in line with their perceived needs [18]. Solid waste management is not always considered a priority, especially in developing countries [19]. Here, tourism drives economic development, and resources are allocated to foster tourist demand rather than reduce its environmental consequences [20].

However, remoteness and size also suggest that tourism stakeholders in small islands know each other better, thus increasing the likelihood of collaboration, especially in critical situations, such as under a threat of environmental degradation. This is explained by the concept of social capital [21] suggesting that individuals and organisations collaborate for survival. This collaboration can be stronger in small and remote areas, such as islands, where social capital is limited and therefore better valued [22]. This is further aligned with the proximity principle in social psychology suggesting that people closer together in a physical environment are more likely to form partnerships than those further away [23]. No empirical research has however been undertaken to understand how/if the concept of social capital and the proximity principle apply to stakeholders in small island destinations where environmental conservation is key to sustain tourism and support resident livelihoods.

This study explores the determinants of stakeholder collaboration for solid waste management in a small island destination through the prism of stakeholder theory, social capital and proximity. Koh Phayam, a small tourism island in Thailand, is used as a case study. Prior to COVID-19, Koh Phayam has been growing in popularity with tourists. This has intensified the challenge of solid waste management calling for urgent mitigative interventions. Stakeholder collaboration is key for the success of these interventions in small tourism islands [16,17,24] and needs to be better understood. The next section provides further theoretical background to the study.

## Literature review

### Stakeholder theory, stakeholder collaboration and social capital

As a theory of organisational management, stakeholder theory posits that organisations have interconnected relationships with different actors of their value chain known as stakeholders [25]. Stakeholders can influence, or be influenced by, organisations; for example, in tourism, stakeholders are represented by customers, employees, or communities hosting tourists [26]. Studying the inter-stakeholder relationships aids in understanding how organisations can more effectively allocate resources to satisfy stakeholders for mutual benefit [27].

Stakeholder theory emphasizes that stakeholders should first be identified and then carefully managed [28]. For example, the problem of overtourism requires tourism organisations to involve destination residents in a dialogue to understand how the problem can be resolved [29]. A successful dialogue improves destination management, which benefits tourism organisations and residents [30]. Stakeholder theory thus enables an understanding of the wider societal and environmental obligations of (tourism) organisations by reinforcing such related concepts as business ethics, corporate social responsibility, and sustainability [31].

Although stakeholder theory does not explicitly integrate the concept of stakeholder collaboration, it communicates the need for stakeholders to shape formal or informal partnerships for common goals [32]. Stakeholder theory is therefore reinforced with a network perspective [33] suggesting that stakeholders should be considered in a network of interdependent partners assisting each other as required [34]. Networks are important for organisations as they enable access to resources, such as finance, labour, and knowledge [35]. Larger and stronger networks improve organisational performance, especially in critical times [36]. For example, strong networks are critical for prompt post-disaster recovery of tourism organisations [37].

Stakeholder theory is underpinned by the concept of social capital [38]. Social capital describes the networks of relationships formed between individuals and/or organisations in a particular society or locality which enable this society or locality to perform more effectively [21]. This suggests that social capital encapsulating the networks of partners is essential for stakeholder collaboration, also in tourism [39]. For example, destinations with stronger tourism stakeholder networks benefit from more effective management and marketing [40]. Social capital, being a key to stakeholder collaboration, is also important for the global transition towards sustainability [41]. The United Nations have recognised this importance in their sustainable development goals whereby Goal 17, Partnerships for the goals, postulates that sustainable development can only be achieved when all stakeholders are involved, and strong stakeholder networks are built [42].

### Stakeholder collaboration for sustainability

Stakeholder collaboration represents a common strategy of working towards sustainability [41]. For example, industrial symbiosis enables organisations to develop a network of partners to foster transition towards the circular economy [43]. Partnerships for sustainability are especially important for tourism where stakeholder collaboration is key for sustainable destination development [16].

Stakeholder collaboration for sustainability is defined as the ability of individuals and organisations, representing private and public sectors, and operating in a specific locality, to work together towards a common, pro-sustainable goal [44]. This goal can be concerned, for example, with environmental conservation, development of local economies, and improvement of residents’ wellbeing [11]. Stakeholder collaboration for sustainability is underpinned by mutual commitment, interest, and trust [45].

The benefits of stakeholder collaboration for sustainability are tangible (for example, improved profitability due to environmental savings) and intangible (for instance, enhanced organisational reputation) [46]. Stakeholders collaborate for sustainability due to personal motives, but also because of pragmatic interests, such as value cocreation [47] or strengthened networks [48]. Stakeholders learn from each other when working in partnership while collaboration contributes to product development and service improvement [45]. Reciprocity is key for successful collaboration as it indicates commitment, facilitates co-learning, and builds trust between stakeholders [49].

Stakeholder collaboration for sustainability is hindered by the lack of stakeholder interest, but also by the limited understanding of the goals and outcomes of collaboration [44]. Stakeholder conflict can occur whereby stakeholders have different visions of the goal, direction, and result of collaboration [50]. The lack and unequal distribution of resources among stakeholders represent another barrier [51]. Linked to this barrier, the imbalance of power can impede stakeholder collaboration for sustainability [52]. The imbalance is exemplified by the situation whereby one stakeholder has more resources and, therefore, more power than others. This can prompt this stakeholder to promote their vision on the extent and direction of collaboration [53]. For example, the imbalance of power has been shown detrimental to stakeholder collaboration for sustainability in former Soviet Union states [54]. Here, public organisations have historically exerted more influence in partnerships, also in the tourism context [55].

Lastly, in tourism, size of a destination plays a role. Larger destinations have more stakeholders; it can therefore be more difficult to engage them in collaboration for sustainability due to varying interests [56]. In this regard, smaller destinations, such as small islands, may be more suited for building successful stakeholder partnerships for sustainability.

### Stakeholder collaboration for sustainable tourism in small islands

The proximity effect is key for inter-organisational networking [57] and successful stakeholder collaboration [58]. Geographical proximity enables stakeholders to interact more effectively which facilitates flows of knowledge, thus creating a better understanding of collaboration goals and building trust [59]. The positive effect of geographical proximity on stakeholder collaboration has been demonstrated in the context of regions where organisations and residents are located close to one another [60,61].

Empirical evidence on the effect of geographical proximity on stakeholder collaboration for sustainable tourism in small islands is fragmented. The study by Graci [16] showcases an example of stakeholder partnership leading to the implementation of innovative sustainability initiatives on the Gili Trawangan island, Indonesia. This study highlights the importance of outlining objectives, defining roles, communicating progress in the implementation of sustainability initiatives, and ensuring equal participation for successful stakeholder collaboration. Arbulú and colleagues [11,17] discuss the opportunities and challenges of stakeholder collaboration for solid waste management in Mallorca, Spain. Similar to the findings of Graci [16], Arbulú and colleagues conclude that partnerships for sustainable tourism development depend upon explicit and equal distribution of roles, costs and benefits. Lastly, Dolezal and Novelli [62] indicate the critical role of stakeholder power and empowerment in building effective partnerships for sustainable tourism development in Bali, Indonesia.

In contrast, Canavan [50] reports an example of unsuccessful stakeholder collaboration for sustainability on the Isle of Man, the UK. Despite geographical proximity, a lack of shared vision and limited trust determine stakeholder exclusion and conflict. Likewise, Towner [32] demonstrate how, despite an enthusiasm to collaborate for sustainable tourism development, stakeholder partnership is limited or avoided in the Mentawai Islands, Indonesia. Corruption, mistrust, and a lack of leadership are identified as the main reasons for unsuccessful collaboration. Lastly, albeit not specifically dealing with failed stakeholder collaboration, Movono and Hughes [63] underline varied stakeholder interests and local culture as the determinants of (in)effective partnerships for sustainable tourism in Fiji.

### Summary of the literature review and the research gap

Literature reiterates that collaboration reinforces the social capital of organisations by extending and strengthening a network of their relationships with stakeholders. The effect of geographical proximity can determine the success of stakeholder collaboration. Stakeholder collaboration is critical for sustainable development of tourism, especially in small island destinations where resources are limited. By pooling together, stakeholders can benefit from each other to enable progress of tourism islands towards sustainability. Research on stakeholder collaboration for sustainable tourism is however limited, especially in developing countries [64]. In particular, there is a paucity of studies on stakeholder collaboration in small islands for solid waste management [17]. This paper aims to partially plug this knowledge gap with a case study of Koh Phayam. The next section introduces the case study destination and explains the research design.

## Materials and methods

### Study area

Koh Phayam is a small island (34.7 km^2^, maximum length 10 km, maximum width 5 km, permanent population of 964) in the Andaman Sea located 33 km off the west coast of Thailand. In 2021, circa 150000 tourists visited the island; this figure was however significantly lower than in the peak year of 2019 when about 400000 tourist arrivals were registered [65]. Given that Thailand recorded a total of 40 million tourists in 2019, this indicates that Koh Phayam accounted for 1% of this volume.

Tourists visit Koh Phayam for sun and beach holidays with the majority (70–80%) arriving from Europe [65]. To reach Koh Phayam, tourists first travel to Ranong, the nearest town and the capital of province, and then by boat to the island. Tourism in Koh Phayam is seasonal: most tourists arrive during the ‘dry’ period between November and April [9]. To accommodate tourist demand, in 2021, there were 92 businesses registered as providers of accommodation and foodservices, including resorts, hotels, hostels, homestays, restaurants and take-aways [65].

Up-to-date figures on the amount of solid waste generated in Koh Phayam do not exist. In 2016, 365 tonnes of solid waste were produced in the island [66]; however, this figure has likely increased since then. Mixed and organic waste were the largest fractions of solid waste in Koh Phayam in 2016 i.e., circa 40% and 30%, respectively, with the bulk of this waste generated by tourism businesses [66]. The organic fraction was represented by food leftovers, and the mixed fraction was composed of packaging waste.

Solid waste in Koh Phayam is collected by four private companies and four non-governmental organisations (NGOs) subcontracted by municipal authorities. These organisations transport solid waste to Ranong for treatment. According to the interview participants (see Findings and discussion), treatment of solid waste from Koh Phayam in Ranong is similar to solid waste treatment in Thailand in general. 32% of solid waste is either reused or recycled; 37% is disposed of, mostly by the methods of landfilling and incineration; and 31% is mismanaged by littering, dumping and open burning [67]. There is no in-situ recycling or recovery facilities in Koh Phayam due to its small size. The management of solid waste in the island is thus ineffective which represents a major challenge in light of anticipated growth in tourism in coastal areas of Thailand in a post-COVID-19 era [68].

### Stakeholder recruitment

The research was approved by the ethics committee of the School of Engineering, King Mongkut’s Institute of Technology Ladkrabang (Bangkok, Thailand). Stakeholders were first identified. These were represented by municipal authorities responsible for environmental conservation and waste management in the island. Private companies and NGOs subcontracted by municipal authorities for solid waste collection and treatment were the other two stakeholder groups. Local communities, represented by community chiefs, were also identified as stakeholders. Lastly, tourism organisations operating in the island i.e., providers of tourist accommodation and foodservices, represented another stakeholder group.

Recruitment of these stakeholders involved multiple stages. At the first stage, contact details of all stakeholders were obtained from public databases and a letter of invitation was produced. This letter introduced the project, explained its aims, and requested a consent for interview. The letter was distributed by email, post and in person. At the second stage, telephone calls were made to all stakeholders who were first contacted by the letter. The calls aimed to confirm that the letter was received and read, reiterate the study’s purpose, and explore stakeholder interest in being interviewed. In the final stage, the stakeholders who preliminarily agreed to be interviewed at the second stage of recruitment, were visited in person. The visits aimed at reconfirming stakeholder intention to become interviewed, explain their rights as interview participants, and answer any final questions related to the study, thus ultimately building trust between prospective participants and the research team.

Following the three-stage recruitment process, 21 stakeholders provided consent to participate in interviews (Table 1). The recruited sample consisted of nine tourism organisations, three representatives of municipal authorities, four environmental NGOs, three private waste collectors, and two community chiefs. It was agreed beforehand that interviews would only involve stakeholder representatives with the decision-making power in the area of interest i.e., solid waste management. For example, in the case of municipal authorities, an interview participant would be a manager directly responsible for environmental conservation. For tourism organisations, it would be a senior manager, such as a resort owner.

**Table 1:** Study participants (n = 26).

Participant code	Gender	Age	Interview duration
TB1	Female	In their 40s	1 h 06 min
TB2	Male	In their 50s	0 h 57 min
TB3	Male	In their 50s	1 h 35 min
TB4	Female	In their 30s	1 h 18 min
TB5	Male	In their 40s	1 h 04 min
TB6	Male	In their 60s	1 h 24 min
TB7	Female	In their 60s	0 h 59 min
TB8	Female	In their 30s	1 h 23 min
TB9	Male	In their 20s	1 h 06 min
G1	Female	In their 40s	1 h 35 min
G2	Male	In their 50s	1 h 07 min
G3	Female	In their 40s	0 h 56 min
N1	Male	In their 30s	1 h 56 min
N2	Female	In their 30s	1 h 35 min
N3	Male	In their 40s	1 h 23 min
N4	Male	In their 30s	1 h 36 min
W1	Male	In their 40s	0 h 58 min
W2	Male	In their 50s	1 h 06 min
W3	Male	In their 40s	1 h 10 min
C1	Male	In their 50s	1 h 36 min
C2	Male	In their 40s	1 h 47 min
T1	Male	In their 40s	1 h 01 min
T2	Female	In their 40s	1 h 03 min
T3	Male	In their 30s	1 h 11 min
T4	Female	In their 30s	1 h 11 min
T5	Female	In their 40s	1 h 23 min

TB—Representative of a business catering for tourists in the island.

G–Representative of a local government office dealing with the issues of waste management and/or environmental conservation.

N–Representative of a non-for-profit organisation dealing with the issues of waste management and/or environmental conservation in the island.

W–Representative of a private sector subcontracted to manage solid waste in the island.

C–Representative of local communities.

T–Tourist.

Besides domestic stakeholders, to gain an ‘external’ perspective on the challenge of solid waste and its management in Koh Phayam, interviews were also conducted with five tourists. These were recruited by convenience sampling during fieldwork. Tourists were approached in the island, explained the project’s aims and invited to partake. Similar to the other study participants, an informed consent was sought from tourists prior to interviewing. No specific requirements were applied to tourist recruitment.

### Data collection

Data were collected by the method of in-depth, semi-structured stakeholder interviews. This method was chosen because of its suitability for exploratory research topics lacking theoretical conceptualisation [69]. This is the case for the current study as the determinants of stakeholder collaboration for solid waste management in small tourism islands are insufficiently understood [17]. The method of semi-structured interviews is also suitable for studying complex social contexts where different actors may have different perspectives on the subject matter in question [70], such as in the case of the determinants of stakeholder collaboration for sustainability [32]. An advantage of semi-structured interviews is their ability to provide detailed insights into a variety of opinions and perceptions on the issues of high societal importance [71], such as solid waste management in small tourism islands, as articulated by different stakeholders [16].

An interview guide was developed based on the literature review. Specifically, the findings reported by Arbulú and colleagues [11,17]; Fuldauer and colleagues [24]; Graci [16]; and Peltola and colleagues [47] were used in the design of interview questions. The guide comprised introductory questions aiming to better understand the current situation with solid waste management in the island, including the amounts of solid waste generated and the logistics behind its collection and treatment. The guide incorporated questions on the roles and responsibilities of different stakeholders alongside the current extent of stakeholder collaboration. Lastly, the guide aimed to explore the desired extent of stakeholder collaboration for solid waste management in the island, covering its determinants. A copy of the interview guide is provided in Supplementary material (S1 Appendix).

The interview guide was developed in English and back translated to Thai. Prior to fieldwork, the interview guide was checked for face and content validity by two academics specialising in environmental conservation and sustainability. The guide was subsequently piloted with four volunteers representing two major stakeholder groups i.e., municipal authorities and tourism businesses. Post pilot, minor modifications were made to the phrasing of some interview questions for better clarity of expression.

Interviews were administered in February-May 2022 and lasted between 56 minutes and 1 hour 56 minutes (Table 1). Prior to interviewing, each prospective study participant was provided with an information sheet explaining the study’s purpose and elucidating the voluntary nature of participation. Participants were subsequently asked to sign and date a consent form.

Interviews were conducted face-to-face in various locations of Koh Phayam and Ranong. Interviews were digitally recorded for transcribing. No financial incentives were provided for participation. Despite the sample of 26 stakeholders, interviews provided rich data showcasing saturation. Although sample size is considered less critical for studies underpinned by qualitative research methods, Marshall and colleagues [72] posit that interview data tend to be saturated within 10–30 datapoints. This current study fits into this recommended datapoint range.

After interviews, subject to consent of the study participants, observations were made by the research team to better understand how solid waste was managed in-situ. For example, in the case of NGOs collecting and treating solid waste, the process of collecting waste on the island, transporting it to Ranong, and treating was observed. In the case of tourism organisations, observations of waste bins were made alongside the process of their collection. This enabled the research team to triangulate the data obtained in stakeholder interviews, thus reconfirming qualitative data, and adding validity to the study’s findings. Data triangulation is considered beneficial and, therefore, highly recommended, for studies of solid waste management [47,73,74].

### Data analysis

**Fig 1:**
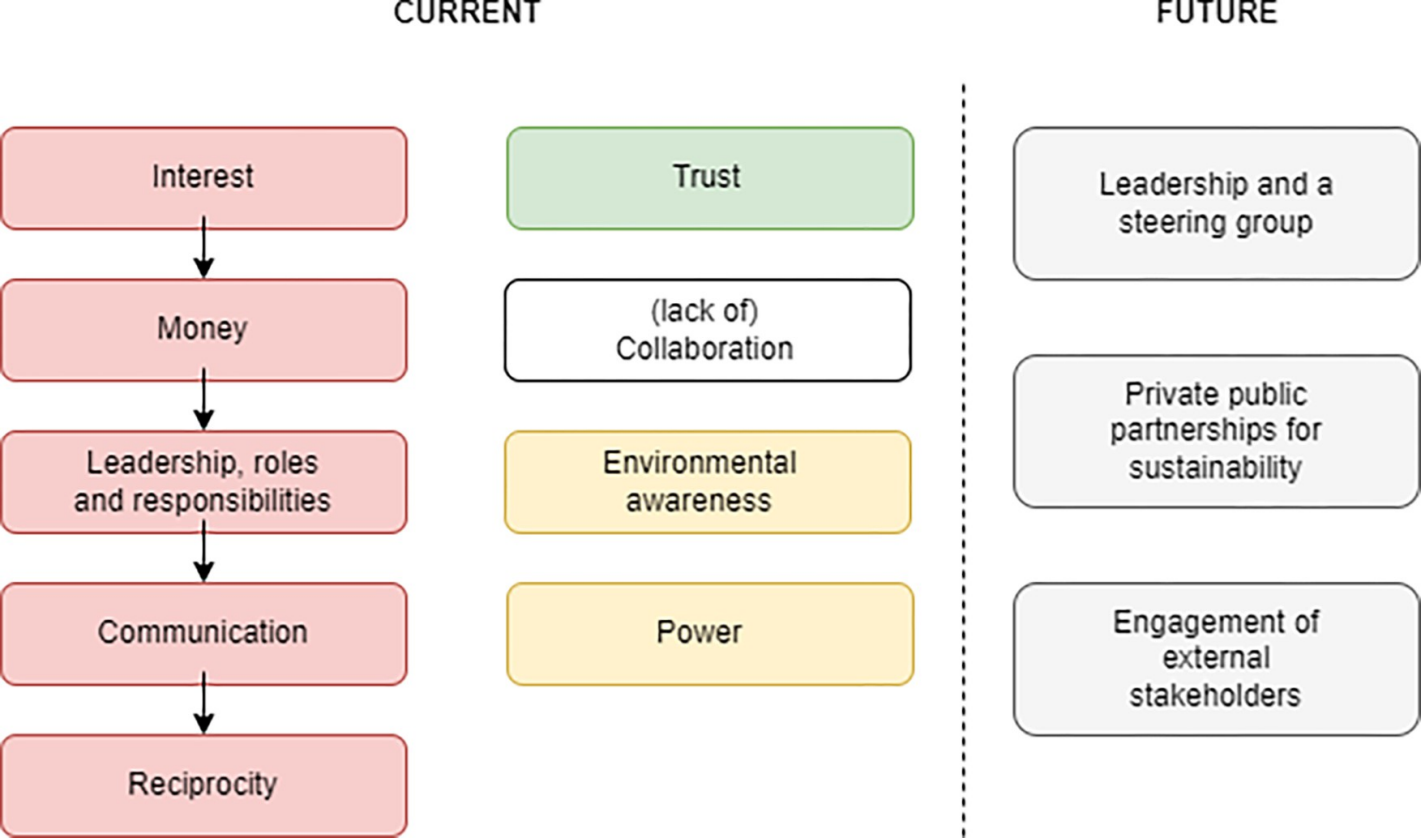
Main codes from thematic analysis. Legend (see in-text explanations for details): Red colour represents the codes with a negative meaning. Yellow colour represents the codes with a neutral meaning. Green colour represents the code with a positive meaning.

Interviews were transcribed verbatim and translated in English by a bilingual member of the research team. Transcribed data were analysed thematically following the guidance by Clarke and colleagues [75]. For trustworthiness in interpretation of meanings, as recommended by Nowell and colleagues [76], data were coded independently by two members of the research team. The outputs of data codification were then compared and any disagreements in interpretation of meanings were discussed until a consensus was achieved. For the write-up of interview findings, exemplar quotes were identified in the transcripts illustrative of the main codes. Fig 1 outlines the scheme of data codification. The next section explains the scheme in detail.

## Findings and discussion

### Solid waste in Koh Phayam: Magnitude and management

The interviews began by establishing the magnitude of solid waste generated in the island and reconfirming the logistics behind its management. Neither solid waste collectors nor municipal authorities were able to provide accurate solid waste figures claiming these to be *‘difficult to get’* (G1). According to one participant, *‘before Corona*, *solid waste was so large in the island that it was simply impossible to measure’* (N2). The lack of accurate figures on solid waste in tourism is a global problem [2] which represents a major challenge from the viewpoint of management. This is because solid waste can only be managed effectively if its magnitude is measured accurately to allocate priority areas for mitigation [77].

Tourism organisations provided solid waste figures based on the weight of waste they generated. Across the sample, total solid waste generation ranged from 5 to 50 kg per day, depending on business size. Mixed waste represented by plastics and glass was the largest fraction (10–30 kg per day) followed by organic waste (5–25 kg per day). The sample average numbers were 18 kg of mixed waste and 14 kg of organic waste generated by a tourism organisation per day. By extrapolating these figures and dividing by the number of tourist arrivals in Koh Phayam in 2019 i.e., 400000, it can be concluded that an average tourist in the island produces circa 2.7 kg of solid waste.

The seminal study by Trung and Kumar [78] suggests that tourists in Vietnam generate, on average, between 2.5 and 7.2 kg of solid waste. A more recent study by Manomaivibool [79] cites the figure of 1.7 kg of solid waste generated by an average tourist in northern Thailand. Given that the current study deals with an island destination, the large amount of solid waste attributed to tourism in Koh Phayam is of major concern.

Data extrapolation also suggests that 92 tourism organisations registered in Koh Phayam generate approximately 1070 tonnes of solid waste per year. Importantly, this figure does not include solid waste produced by households. If households are added, the total amount of solid waste generated in the island will be even more significant. However, even the smaller amount attributed to tourism businesses is three-fold more than the figure of 365 tonnes of solid waste recorded for Koh Phayam in 2016 [66]. Similar to the conclusion by Arbulú and colleagues [11], this demonstrates how rapid growth of tourism in the island contributes to the increase in solid waste generation, thus calling for urgent mitigation via stakeholder collaboration.

### Stakeholder collaboration for solid waste management: Current state

#### Barriers

All stakeholders recognised the problem of solid waste in the island as significant; collaboration for its effective management was however limited. Municipal authorities worked with solid waste collectors, but this collaboration, despite being formal and contractually bound, was caused *‘by necessity rather than free will’* (G2). Solid waste collectors partnered with some tourism organisations and local communities from where they collected the mixed fraction of solid waste. However, these partnerships were not formalised. Overall, all stakeholders agreed that collaboration could be improved by showcasing its benefits to the parties involved:

‘*I don’t work with anybody [to manage solid waste]*, *there is little sense*. *I pay 30 Baht per month [0*.*9 USD] for [waste] collection*. *Even if I don’t pay*, *they [solid waste collectors] still come and collect the rubbish*. *I don’t think it [collaboration] is that important given the money involved is ridiculous…’* (TB2)

This quote highlights a lack of financial incentives as one of the barriers for more effective stakeholder collaboration (Fig 1). Collection changes are too small for tourism organisations to become interested in separating solid waste in-situ for profit. Solid waste collectors pay between 1 to 11 Bahts [0.03–0.3 USD) per 1 kg of separated plastics. Tourism organisations, and even island residents, do not consider it worth to separate solid waste for such small payment. Concurrently, solid waste contractors either do not get paid by municipal authorities or this payment is very small. This forces solid waste collectors to only offer a small fee in return for separated plastics. Further, solid waste collectors deliver mixed waste to Ranong for extracting recyclables, such as metal, glass and plastics, for subsequent re-sale. Organic waste cannot be re-sold, and it is therefore not collected. As a result, food leftovers are either dumped by tourism organisations in the sea or disposed of by the method of land spreading. Lastly, given there are eight companies collecting solid waste in Koh Phayam, imposing a higher collection charge on tourism organisations and island residents is not considered viable because of competition.

Evidence from developed countries shows that carefully designed financial incentives can be instrumental in promoting recycling and, ultimately, stakeholder collaboration for solid waste management [80]. Even organic waste can be resold to produce compost or pet food [43]. A lack of financial incentives in Koh Phayam determines stakeholder disinterest in partnering for sustainability. To make stakeholders interested, a more effective pricing mechanism should be developed. For example, payments to solid waste collectors by municipal authorities can be increased, and a ‘pay-as-you-throw’ scheme can be introduced for mixed waste to incentivise tourism organisations for in-situ solid waste separation and recycling [9].

A lack of leadership and limited understanding of stakeholder roles and responsibilities was another barrier (Fig 1). Tourism organisations and local residents blamed municipal authorities for not leading on solid waste management while municipal authorities expected tourism organisations to be more proactive and take the lead. For example, given that organic waste is primarily disposed of by dumping in the sea, municipal authorities requested, but never reinforced, in-situ separation of the organic fraction. However, they did not provide a separate bin for this purpose assuming it would be obtained by tourism organisations and local residents. Although NGOs provided such bins at a later stage, they did not explain how to use them for separation:

‘*They [municipal authorities] told us to separate food from other waste*. *OK*, *I said*, *but where to*? *They [municipal authorities] said*, *oh*, *get your own bin*. *I said*, *why should I if I can dump it*?.. *I tried to compost food waste*. *I got one of those composter things*. *It was my own idea*, *no one helped me*, *no one told me what to do and how to do it*. *But then I had to stop*. *There is no space*, *and the odour is horrendous…’* (TB7)

Different expectations of leadership i.e., ‘bottom up’ versus ‘top down’, and poor assignment of roles and responsibilities hinder stakeholder collaboration for sustainability [81]. To minimise this hindrance, one stakeholder should take the lead while other stakeholders need to accept this leadership and follow the roles assigned [64]. In developing and transition economies public sector organisations i.e., municipal authorities in the current study, often choose to lead on sustainability projects [55]. However, if a public sector organisation takes the lead, it is critical to ensure it follows so-called *‘responsible leadership’* in that, for example, there is no corruption and unequal treatment involved [82]. To achieve this, a leader’s decisions and activities should be overseen by other stakeholders.

Communication was another issue (Fig 1). Stakeholders did not always inform each other about sustainability projects they would like to develop. For instance, municipal authorities considered an option of building an incinerator in Ranong to treat the growing amount of solid waste from the island. This option was not however communicated to solid waste collectors and tourism organisations although some of the latter were even prepared to co-finance an incinerator, subject to its adequate cost. Likewise, some tourism organisations requested solid waste collectors to take organic waste from the island for composting in Ranong. No definite answer was received and, hence, organic waste continued to be treated either by dumping or land spreading:

‘*It’s a small island but we don’t speak to each other which*, *in my opinion*, *is a key problem*. *For example*, *there was a Swiss company willing to buy waste from the island*. *They approached XXX [name removed for anonymity*, *an NGO representative] with a proposal*. *I don’t know what happened*, *but the contract was never signed*. *I think they should have told us about why they’d decided to turn them down’* (C1)

Communication is key for stakeholder collaboration for sustainability, especially in developing and transition economies [83] and should therefore be reinforced in Koh Phayam. Regular meetings or round tables can be organised to update stakeholders on progress in solid waste management and discuss plans and proposals. These meetings can be conducted online to save on travel time and facilitate participation.

Reciprocity was another barrier (Fig 1). Some stakeholders committed more to solid waste management than others. For example, a restaurant owner started offering a free drink to tourists coming with refillable bottles. They proposed the idea to other restaurants, but these did not take it. Likewise, a resort owner proposed to solid waste collectors and municipal authorities an idea of constructing a facility converting organic waste into biofuel. However, the idea was rejected with no reason given. Lastly, a hotel owner suggested composting to a local community which was, however, not pursued:

‘*I went to local villagers and said*, *hey*, *how about I bring you organic waste and you compost here as you have more space than I do*. *But they say*, *oh*, *no*, *we don’t want that as it stinks*. *So*, *this is how it goes here in terms of reciprocity [laughter]’* (TB4)

Reciprocal engagement is key for success of stakeholder partnerships for sustainability [49]. In Koh Phayam it can be achieved via regular capacity building events offering the opportunity for stakeholders to discuss the ideas of more effective solid waste management and agree on the extent of their involvement in the implementation of these ideas [44]. Reciprocity can also be achieved via more effective communication which should explicitly articulate the benefits of collaboration to all involved [84].

#### Enablers

As for enablers of stakeholder collaboration, trust was frequently mentioned (Fig 1). Although the literature forewarns that mistrust can impede formation of partnerships [32], this was not the case for Koh Phayam. The study participants had no concerns over each other, referring to small size of the island as a positive factor in building social capital. The current lack of collaboration was attributed to other factors, as discussed above. Studies conducted in the context of Thailand and its sustainable tourism industry suggest that local stakeholders tend to trust each other which can be partially attributed to the national Thai culture [85,86]. This current study adds further evidence to this point:

‘*It’s a small island and we know each other*. *How can I say that I don’t trust XXX [name removed for anonymity*, *a representative of solid waste collectors]*, *if I see him every week in the market*? *It’s not about trust*, *it’s about other things…’* (N3)

Power was discussed as an enabling factor from a neutral perspective (Fig 1). There was no particular stakeholder with more power than others. Further, the study participants claimed they had enough power to manage solid waste within their own organisations. For example, organic waste was never a problem for one resort owner as they claimed to use it for in-garden composting. However, when considering the island as a whole, this was where the stakeholders felt powerless to improve solid waste management. In other words, while individual power was available, there was a lack of cumulative, multistakeholder power.

The literature emphasizes power as a determinant of stakeholder collaboration for sustainability. However, power is largely discussed from the perspective of its imbalance [87] or even abuse [88]. This current study adds to the body of knowledge by indicating a new notion of power i.e., *‘powerful as individuals but powerless when together’* (TB5). To reduce the negative effect of this ‘powerlessness in togetherness’, capacity building events and round tables are warranted. Such events can empower stakeholders by reinforcing their beliefs in that a success is achievable when working in partnerships. Such events can also strengthen perceived reciprocity i.e., another determinant of stakeholder collaboration, as highlighted earlier.

Environmental awareness was discussed as an enabler (Fig 1) with the study participants claiming that it was high among tourists, but low among residents. For instance, a solid waste collector complained that local residents would often contaminate recyclables with the organic fraction although it was repeatedly emphasized to them that contamination hindered recycling and recovery. In contrast, a resort owner praised tourists for refusing to accept single-use plastic straws and cups. This was confirmed by one of the tourists:

‘*It’s a beautiful island*, *almost untouched*, *you know*. *But I was shocked when I saw so much garbage here*. *I understand this is very much from tourism*, *but I feel the locals don’t try to protect the environment either*. *The bins are provided for plastics*, *but it is felt that only tourists are using these bins’* (T3)

Stakeholder collaboration for sustainability should be underpinned by an understanding of why this collaboration is necessary and what it aims to achieve [44]. In Koh Phayam some stakeholders lack this understanding. To raise environmental awareness of island residents and tourists, education campaigns are necessitated. These should be designed and implemented by municipal authorities, ideally with the involvement of educational institutions, such as schools, but also by tourism organisations. A system of clear incentives should be developed to encourage environmental conservation, such as free drinks for refillable bottles, as discussed above.

Importantly, the barriers and enablers of stakeholder collaboration for sustainability are inter-related. For example, by building and reinforcing trust, stakeholders can reduce such barriers as restricted leadership and limited communication [89]. Likewise, the balance of power can strengthen reciprocal relationships and increase stakeholder interest in collaborating with one another [55]. Therefore, facilitating conditions need to be created to ensure the enablers of stakeholder collaboration for sustainability are reinforced in Koh Phayam.

When comparing the findings of the current study with the literature on stakeholder collaboration for sustainability in small tourism islands, some similarities and differences can be established. Similar to Graci [16], the current study showcases leadership and communication as key for successful stakeholder collaboration for sustainable tourism in islands. In line with the findings reported by Arbulú and colleagues [11,17], the current study demonstrates the importance of distributing and accepting roles and responsibilities among stakeholders for effective collaborative work towards solid waste minimization. Lastly, similar to Dolezal and Novelli [62], the current study highlights power as a critical enabler of pro-sustainability, multi-stakeholder collaborative projects. However, unlike Towner [32], Canavan [50] and Movono and Hughes [63] who all report how limited trust can alienate stakeholder collaboration in small islands, the current study shows that geographical proximity in Koh Phayam is important for building and reinforcing trust, thus improving chances for local stakeholders to work together towards more effective solid waste management. This suggests that local context plays a critical role when evaluating the potential of stakeholder collaboration for sustainability in small tourism islands, thus highlighting the need for more focal, localised studies.

### Future vision

The study participants outlined three determinants of successful stakeholder collaboration for future management of solid waste in Koh Phayam (Fig 1). First, leadership was considered essential, and the majority agreed that municipal authorities were best positioned to lead. However, to avoid accusations in corruption, a steering committee was proposed. The role of this committee, composed of the representatives of all stakeholder groups, would be to oversee activities of the leading stakeholder and intervene as necessary. Wondirad and colleagues [64] argue that steering committees are especially important for stakeholder collaboration in developing economies given their immature traditions of pro-environmental governance.

Private public partnerships (PPPs) for sustainability were discussed (Fig 1). Although the study participants did not necessarily know these by name, the arrangements which they envisaged i.e., long-term agreements between public and private organisations delivering solid waste management projects, contained explicit characteristics of PPPs. Although the current structure of solid waste management in Koh Phayam is underpinned by the PPP idea, it does not function as PPP. PPPs should be based upon a strategic vision, assessment of available resources, and evaluation of required investments [11]. These PPP features can facilitate adequate market pricing for solid waste collection and treatment; they can also enable the design of effective financial incentives for in-situ separation and recycling [17]. Given that trust, as a key element of effective stakeholder collaboration, already exists in Koh Phayam, implementation of PPPs should be considered. As a start, capacity building events and round tables should be organised to mobilise prospective participants and establish their interests, aims and expectations.

Lastly, potential engagement of external stakeholders in solid waste management in Koh Phayam was discussed (Fig 1). There is no large-scale agricultural production in the island, but the amount of organic waste is significant; hence, a proposal was made to engage farmers in Ranong in solid waste management. Currently, organic waste is either dumped in the sea or used for on-site composting. Instead, organic waste can be transported from the island to the hinterland and either composted on local farms or used for production of biofuel. This network of interconnected organisations is commonly known as the industrial symbiosis [43], and it can be implemented in Ranong in the form of, for example, community-scale composting. Community-scale composting can be operated with as little as 200 kg of organic waste per day, or 73 tonnes per year [90]. The amount of organic waste generated in Koh Phayam (1070 tonnes per year) is sufficient to meet this requirement. Organic waste will however need to be properly separated in-situ which underlines the importance of stakeholder commitment and emphasizes the role of financial incentives in reinforcing stakeholder participation:

‘*Ideally*, *I’d want my food waste collected every day*. *Preferably*, *for free but I’d even be happy to give it out for a small fee*. *If this waste is then delivered to a farmer*, *I think that is ideal*. *No dumping in the sea*, *no unpleasant smell*, *yeah*, *I’d feel good about it [laughter]’* (TB3)

## Conclusion

This study explored the opportunities and challenges of stakeholder collaboration for solid waste management in a small tourism island, thus reinforcing the emerging body of literature on partnerships for sustainability. Through the lens of stakeholder theory, social capital and proximity principle, the study revealed such impediments of stakeholder collaboration for solid waste management in Koh Phayam as a lack of perceived benefits, leadership, communication, and reciprocity. The study indicated that geographical proximity exerted a positive effect on social capital and trust between stakeholders. It also ensured that disbalance of power, a major barrier to partnerships for sustainability in developing countries, was less pronounced in the case studied island.

This finding suggested sufficient grounds for future stakeholder collaboration for solid waste management in Koh Phayam, subject to addressing other barriers, as highlighted above. From the theoretical perspective, the study contributed to a better understanding of geographical proximity as a determinant of effective partnerships for sustainability. This finding is relevant for small tourism islands, but it can also be extended to other tourism contexts, such as remote mountain destinations, characterised by a small number of stakeholders concentrated in a single locality.

From the management perspective, the study highlighted several areas for intervention which can facilitate stakeholder collaboration for solid waste management in Koh Phayam. Financial incentives need to be designed to encourage stakeholder engagement in solid waste management. Capacity building workshops and round tables can streamline stakeholder communication and facilitate reciprocity. One stakeholder, most likely municipal authorities, should take the lead in solid waste management and a steering committee should be established to oversee a leader’s activities. The feasibility of setting PPPs for solid waste management in Koh Phayam should be considered given that local stakeholders know and trust each other. Lastly, external stakeholders can be involved in management of organic waste, thus extending partnerships for sustainability towards other localities.

The study had limitations which outlined directions for future research. First, not all stakeholders in Koh Phayam participated in interviews. Future research should aim at engaging other stakeholders and examine their opinions either by a method of interviews, or by focus groups and surveys. Second, the study did not incorporate stakeholders external to Koh Phayam and Ranong, such as farmers and national decision-makers. As these stakeholders are critical for the design of financial incentives for solid waste management alongside PPPs and industrial symbiosis networks, their opinions should be sought in future research. Third, this study established that organic waste in Koh Phayam was severely mismanaged. Future research should focus on organic waste as a key challenge in small tourism islands and examine how it can be managed more effectively. Fourth, the idea of building PPPs for solid waste management in Koh Phayam emerged during interviews. PPPs can be instrumental to progress small tourism islands towards sustainability; however, research on how they can be designed and implemented remains limited, especially in countries of the Global South. Hence, more studies are necessitated to better understand how PPPs can be most effectively developed in Koh Phayam or in other island destinations of Thailand to account for local political and socio-economic contexts. Lastly, this study was only concerned with Koh Phayam. However, Thailand has numerous small tourism islands, including the world-famous destinations of Phuket, Koh Samui and Koh Phi Phi. Future research should focus on these islands as they represent the mainstay of tourist demand.

## Supporting information

S1 Appendix Interview guide. (DOCX)
