# Supplementary material for: Stakeholder collaboration for solid waste management in a small tourism island
Source: PLoS One. 2023 Jul 26;18(7):e0288839. doi: 10.1371/journal.pone.0288839 (PMC10370731; doi:10.1371/journal.pone.0288839)
Supplement: S1 Appendix — (DOCX) [file pone.0288839.s001.docx]

**Appendix 1. Interview guide.**

Introduce yourself

Explain the purpose

Reconfirm willing to participate

Sign consent form

Introductory questions:

- Tell me about your organisation and your role within your organisation
- Now tell me about solid waste in Ko Phayam
  - *How much is generated?*
  - *What are the main fractions?*
  - *Who generates the most?*

Main questions:

- Now tell me how, in your opinion, solid waste is managed in Ko Phayam
  - *Who is responsible for what? What is your responsibility in it?*
  - *What works well and what does not?*
  - *What are the reasons?*
- Now tell me if you collaborate with any other organisation(s) in Ko Phayam or Ranong for solid waste management
  - *Which organisations do they know? Which ones do you work with?*
    - *Who takes the lead and why?*
  - *Why do you (not) work together?*
  - *What is the role of power?*
  - *What conflicts are there, if any?*
  - *How reciprocal is this collaboration, if at all?*
  - *What is the role of resources?*
    - *Labour, money, knowledge*
- Now tell me how this collaboration can be improved?
  - *What are the main barriers for more effective collaboration?*
  - *How can these be resolved?*
  - *Who should be in charge?*
  - *What are you prepared to do in your organisation?*
- Now describe to me an ideal situation in terms of solid waste management in Ko Phayam, in your opinion
  - *How do you envisage it?*
  - *What or who is instrumental to making it work?*
  - *What is the role of collaboration with other stakeholders?*
  - *Are there examples of effective solid waste management and stakeholder partnerships from other localities which you may be familiar with and would like to share?*

Any other thoughts, not covered above?

Thank you!
